# Supplementary material for: COVID-19 Infection Fatality Rate Associated with Incidence—A Population-Level Analysis of 19 Spanish Autonomous Communities
Source: Biology (Basel). 2020 Jun 16;9(6):128. doi: 10.3390/biology9060128 (PMC7345771; doi:10.3390/biology9060128)
Supplement: Supplementary file 1 [file biology-09-00128-s001.pdf]

Supplementary Online File

Methods 2

Table S1. Total number of COVID-19 attributed deaths (N Deaths), Infection fatality rate (IFR), case fatality rate (CFR), seroprevalence of SARS-CoV-2 (Sero-Prev), cumulative number of cases of COVID-19/100,000 (Cases), COVID-19 attributable mortality /100,000 (Mortality), COVID-19 attributable hospitalization/100,000 (Hospital) in 19 Spanish regions and percent 65 years old or older (Age 65). 4

Table S2: Multiple linear regressions of associations between infection fatality rate and case fatality rate, mortality/100,000, cases/100,000, hospitalizations/100,000 and seroprevalence, controlling for percent of the population 65 years or older. 5

Table S3: Sensitivity analysis of associations between infection fatality rate and case fatality rate, mortality/100,000, cases/100,000, hospitalizations/100,000 and seroprevalence excluding 2 regions/cities with total number of deaths under 100 6

Methods

Dependent variable:

The *infection fatality rate* per region was calculated as the percent of individuals who died from COVID-19 as of 13/5/2020 (source: [1]) divided by the percent of individuals seropositive for COVID-19 in the seroprevalence survey (source: [2])

Independent variables:

The *cumulative number of confirmed cases of COVID-19 infection per 100 000* inhabitants on 13/5/2020 per region. The number of confirmed cases was ascertained by a number of diagnostic methodologies including PCR and serology [1]. This data was obtained from a Spanish Ministry of Health epidemiology report [1].

The *hospitalizations related to COVID-19 infection per 100 000 inhabitants* on 13/5/2020 per region. This data was obtained from a Spanish Ministry of Health epidemiology report [1].

The *COVID-19 mortality rate per 100 000 inhabitants* on 13/5/2020 per region. This data was obtained from a Spanish Ministry of Health epidemiology report [1].

The crude *case fatality rate per region* was calculated as the deaths related to COVID-19 divided by the total number of COVID-19 cases multiplied by 100.

The *percent aged 65 years old or older* per region was defined as the percent of the regional population that was 65 years old or older in 2019. This data was taken from the Spanish National Institute for Statistics:

https://www.ine.es/jaxiT3/Datos.htm?t=1451#!tabs-tabla

The estimated population sizes of each region for 2019 were obtained from Wikipedia: https://simple.wikipedia.org/wiki/Ranked_list_of_Spanish_autonomous_communities_by_population

Data analysis

Spearman's correlation was used to assess the association between regional IFR and CFR, seroprevalence of SARS-CoV-2, incidence of COVID-19 cases/100,000, COVID-19 mortality/100,000 and COVID-19 hospitalizations/100,000. A sensitivity analysis was conducted in which these correlations were repeated controlling for the percent of the population aged 65 years or older. Linear regression was used for these analyses. The analysis was performed in STATA version 16 (Stata Corp, College Station, Tx).

Table S1. Total number of COVID-19 attributed deaths (N Deaths), Infection fatality rate (IFR), case fatality rate (CFR), seroprevalence of SARS-CoV-2 (Sero-Prev), cumulative number of cases of COVID-19/100,000 (Cases), COVID-19 attributable mortality /100,000 (Mortality), COVID-19 attributable hospitalization/100,000 (Hospital) in 19 Spanish regions and percent 65 years old or older (Age 65).

Region	N Deaths	IFR (%)	CFR (%)	Sero-Prev. (%)	Cases	Mortality	Hospital	Age 65 yo (%)
**Andalucía**	1332	0.6	10.8	2.7	185.0	15.8	72.4	17.16
**Aragón**	829	1.3	15.4	4.9	508.1	61.4	193.7	21.65
**Asturias**	307	1.6	13.0	1.8	301.8	28.5	207.8	25.66
**Baleares**	215	0.8	11.0	2.4	184.3	19.2	101.1	15.82
**C. Valenciana**	1341	1.0	12.5	2.5	273.8	26.1	104.7	19.51
**Canarias**	151	0.4	6.6	1.8	107.2	7.1	44.2	16.22
**Cantabria**	205	1.1	9.1	3.2	460.2	34.5	173.3	21.9
**Castilla La Mancha**	2835	1.2	17.2	10.8	1139.8	133.6	422.4	18.99
**Castilla y León**	1919	1.0	10.6	7.2	952.9	75.4	334.5	25.23
**Cataluña**	5692	1.3	10.3	5.9	751.9	75.2	386.6	19.12
**Ceuta**	4	0.4	3.5	1.1	205.9	4.8	13.1	12.02
**Extremadura**	27104	1.5	16.8	3	353.7	44.1	158.3	20.64
**Galicia**	489	1.0	6.4	2.1	387.6	21.5	104.4	25.16
**La Rioja**	597	3.3	8.7	3.3	1667.8	107.5	457.7	21.11
**Madrid**	348	1.2	13.4	11.3	1080.7	134.8	644.1	17.86
**Melilla**	8760	0.1	1.7	1.9	163.4	2.5	54.5	10.72
**Murcia**	2	0.7	9.3	1.4	162.6	9.6	45.2	15.78
**Navarra**	142	1.3	9.7	5.8	1202.7	76.6	316.8	19.8
**País Vasco**	494	1.6	10.9	4	825.1	65.8	317.1	22.6

Table S2: Multiple linear regressions of associations between infection fatality rate and case fatality rate, mortality/100,000, cases/100,000, hospitalizations/100,000 and seroprevalence, controlling for percent of the population 65 years or older.

	CFR Model		Mortality Model		Cases Model		Hospitalization Model		Seroprevalence Model	
	Coeff. (95% CI)	P-value	Coeff. (95% CI)	P-value	Coeff. (95% CI)	P-value	Coeff. (95% CI)	P-value	Coeff. (95% CI)	P-value
65 years & older (%)	.0008 (.0000-.0015)	0.047	.0006 (.0000-.0013)	0.042	.0005 (.00001-.00107)	0.046	.0006 (.000009-.001)	0.021	.0009 (.0002-.0016)	0.019
CFR	.00023 (-.0006-.0010)	0.553	NA	NA	NA	NA	NA	NA	NA	NA
Mortality	NA	NA	.00007 (.0000-.0001)	0.020	NA	NA	NA	NA	NA	NA
Cases	NA	NA	NA	NA	.000009 (.000004-.00001)	0.001	NA	NA	NA	NA
Hospitalization	NA	NA	NA	NA	NA	NA	.00002 (.000003-.00003)	0.050	NA	NA
Seroprevalence (%)	NA	NA	NA	NA	NA	NA	NA	NA	.0002 (-.0008-.0012)	0.629

Table S3: Sensitivity analysis of associations between infection fatality rate and case fatality rate, mortality/100,000, cases/100,000, hospitalizations/100,000 and seroprevalence excluding 2 regions/cities with total number of deaths under 100

	Spearman rho	P-value
CFR	0.29	0.2500
Mortality	0.68	0.0025
Cases	0.68	0.0026
Hospitalization	0.72	0.0012
Seroprevalence	0.42	0.0896

References

1. Ministry of Health. Actualización nº 104. Enfermedad por el coronavirus (COVID-19). 13.05.2020. https://wwwmscbsgobes/profesionales/saludPublica/ccayes/alertasActual/nCov-China/documentos/Actualizacion_104_COVID-19pdf. 2020.
2. MInistry of Health. Estudio ENE-COVID-19: Primera ronda estudio nacional de sero-epidemiologia de la infeccion por SARS-CoV-2 in Espana. https://wwwcienciagobes/stfls/MICINN/Ministerio/FICHEROS/ENECOVID_Informe_preliminar_cierre_primera_ronda_13Mayo2020pdf. 2020.
